# Pembrolizumab-Induced Sarcoid-Like Reaction in a Patient With Lung Cancer

**DOI:** 10.7759/cureus.12395

**Published:** 2020-12-31

**Authors:** Hira Yousuf, Rasheid Mekki, Khizer Khan, Ali Hussain

**Affiliations:** 1 Oncology, Pinderfields General Hospital, Wakefield, GBR; 2 Medical Oncology, Pinderfields General Hospital, Wakefield, GBR; 3 Respiratory Medicine, Pinderfields Hospital, Wakefield, GBR; 4 Acute Medicine, Pinderfields General Hospital, Wakefield, GBR

**Keywords:** sarcoid like reaction, pembrolizumab, lung cancer, case report, programmed cell death protein 1, tumour necrosis factor-α (tnf-α), heerfordet syndrome

## Abstract

Cancer is not only the leading cause of mortality and morbidity but also poses a major economic burden. Until recently, onco-immunotherapy has dramatically changed the landscape of cancer treatment. For example, an inhibitor of programmed cell death (PD-1) plays a vital role by potentiating effective immune-mediated destruction of tumor cells. However, the spectrum of immune-related adverse events especially granulomatous sarcoid lesions has been recognized too. Such lesions involve the dermis and subcutaneous tissue (panniculitis) and lymph nodes. Herein we are presenting a case report of a patient, who developed a sarcoid-like reaction after treatment with pembrolizumab.

## Introduction

Pembrolizumab is a novel humanized monoclonal antibody that targets programmed cell death protein 1 (PD-1) cell signaling. PD-1 is a checkpoint protein on T cells where it normally acts as a type of “off switch” which prevent T cells from attacking other cells in the body. Some cancer cells produce a large amount of programmed death-ligand 1 (PD-L1) and use it as an escape mechanism from an immune attack. Pembrolizumab negates such an escape mechanism and thus restores anti-tumor-cell response against tumor cells. However, preventing the inhibition of T cell activation results in both immune and inflammatory reactions. Granulomatous/sarcoid-like lesions (clinical or radiological) are recognized as toxicity associated with pembrolizumab. Pembrolizumab-related lesions exhibit sarcoid granuloma on histopathological sections. Early recognition of such side effects is important as they often mimic disease recurrence and/or consequently may lead to cessation of therapy.

## Case presentation

A 62-year-old female with a past medical history of hypertension and ex-smoker (> 50 pack-years) was diagnosed with T1C, N0, M0, adenocarcinoma of the right lung and underwent right video-assisted thoracoscopic surgery (VATS). Subsequent CT chest showed relapse of disease with progressive mediastinal, right hilar, and subcarinal lymphadenopathy. Furthermore, her biomarkers programmed death-ligand 1 (PD-L1) was positive (100%), epidermal growth factor receptor (EGFR), anaplastic lymphoma kinase (ALK) wild type (negative), and no ROS proto-oncogene 1 (ROS-1) translocation. Given the high level of PD-L1 positivity, she was placed on pembrolizumab 200 mg intravenously every three weeks.

After nine weeks of treatment, she developed right wrist pain and hyperpigmentation of her previous scars (surgical scars-episiotomy and accidental scars). Additionally, she also reported some new skin lesions on her face, especially involving nasolabial groove and noticed subcutaneous thickening on the right forearm. Ultrasound of forearm revealed an ill-defined subcutaneous abnormality for a length of about 9.5 cm extending from the ulnar aspect of the wrist to the level of mid-forearm. It showed extensive subcutaneous edema, ill-defined hyperechoic areas with marked vascularity.

**Figure 1:**
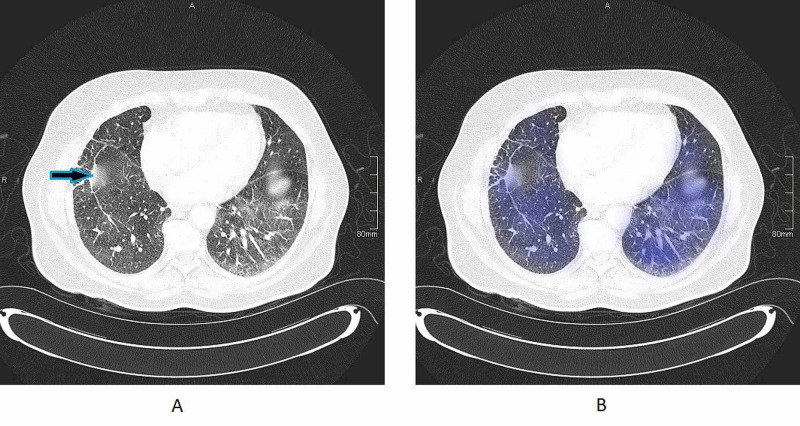
A shows bilateral ground glass appearance with arrowhead showing old surgical scar. B shows shaded areas which explain areas of ground glass changes.

Physical examination revealed sclerotic scars on bilateral shins. Additionally, face examination revealed an erythematous palpable dermal change to a small scar on the right cheek, numerous erythematous papules to the left cheek which are not related to previous scars. Angiotensin-converting enzyme (ACE) was 116 (normal range 8-52 IU/L) and erythrocyte sedimentation rate (ESR) was 7 (normal range 1-14 mm/h). Polymerase chain reaction (PCR) for Mycobacterium tuberculosis and Ziehl-Neelsen (ZN) staining were negative. CT scan thorax (Figure 1A,B) revealed ground glass changes more marked on left lower lobe.

**Figure 2:**
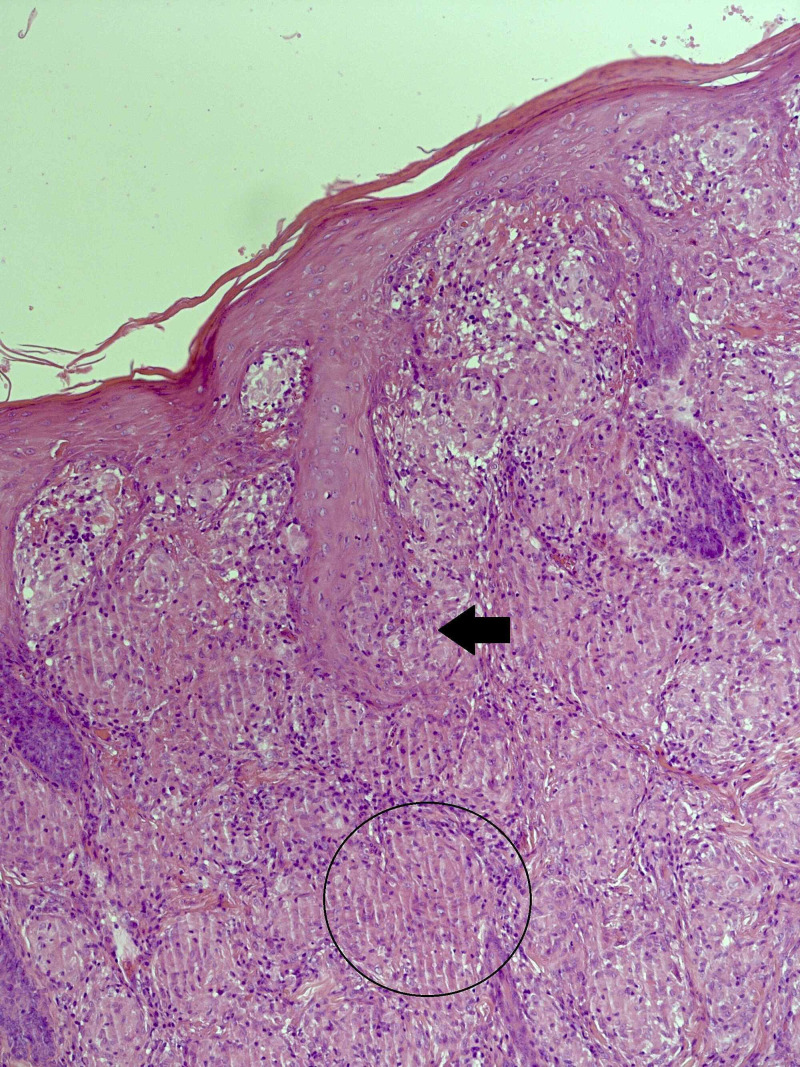
A section of skin stained with H&E. The dermis is packed with relatively well-defined non-necrotizing granulomata, some of which are surrounded by a loose cuff of lymphocytes (circled area). The skin adnexae are involved and disrupted by the granulomata (black arrow). H&E, hematoxylin and eosin

**Figure 3:**
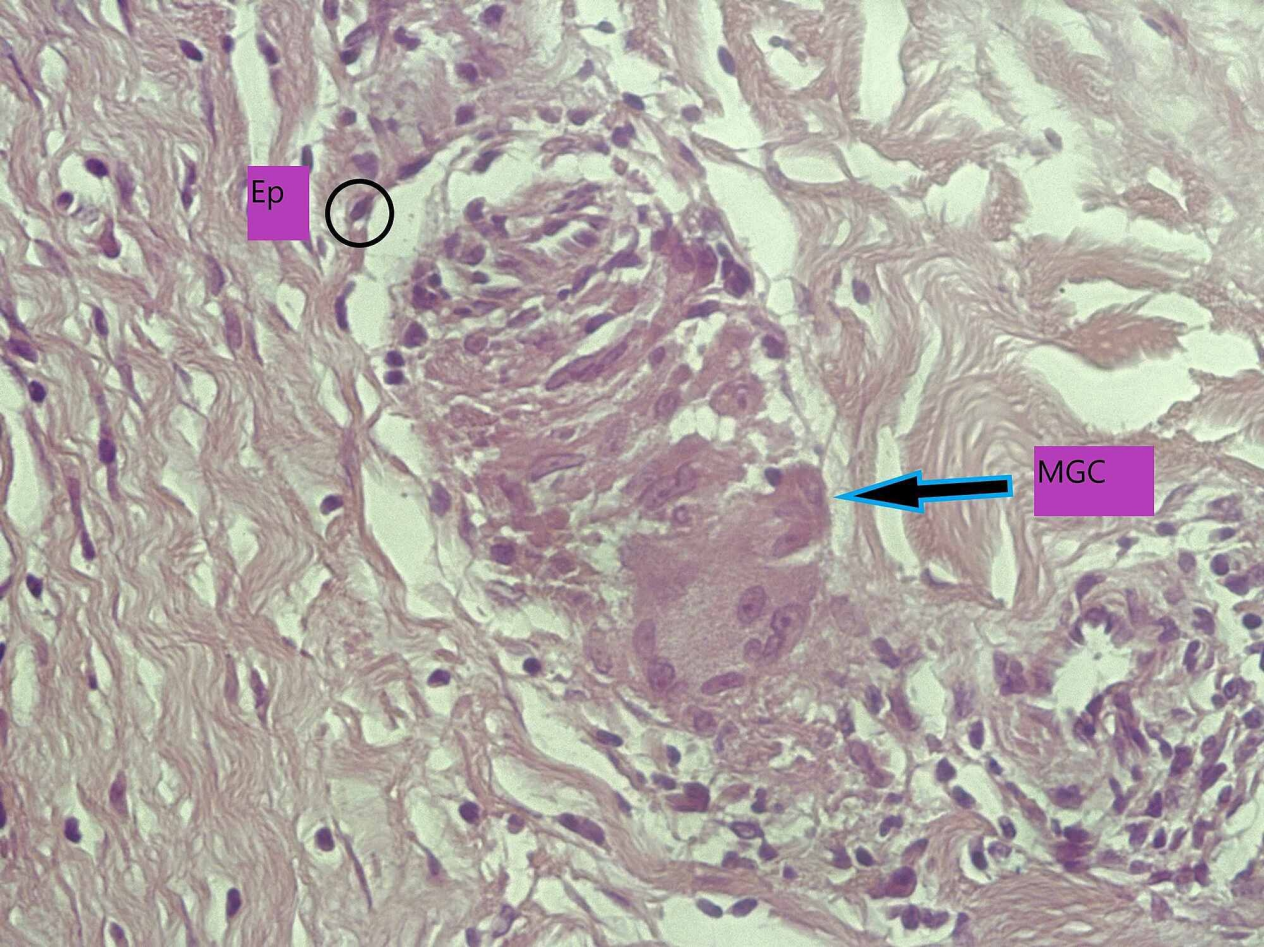
A high-power image of a non-necrotizing granuloma (H&E), showing epithelioid histiocytes (circle) and admixed multinucleated giant cell (arrow). Ep, epitheiod histocyte; MGA, multinucleated giant cells; H&E, hematoxylin and eosin

Skin punch biopsies (Figures 2-3) performed from cheek and shins showed plentiful non-necrotizing granulomas within the dermis, along with a small number of admixed lymphocytes and giant cells. There is no evidence of atypia or malignancy.

**Figure 4:**
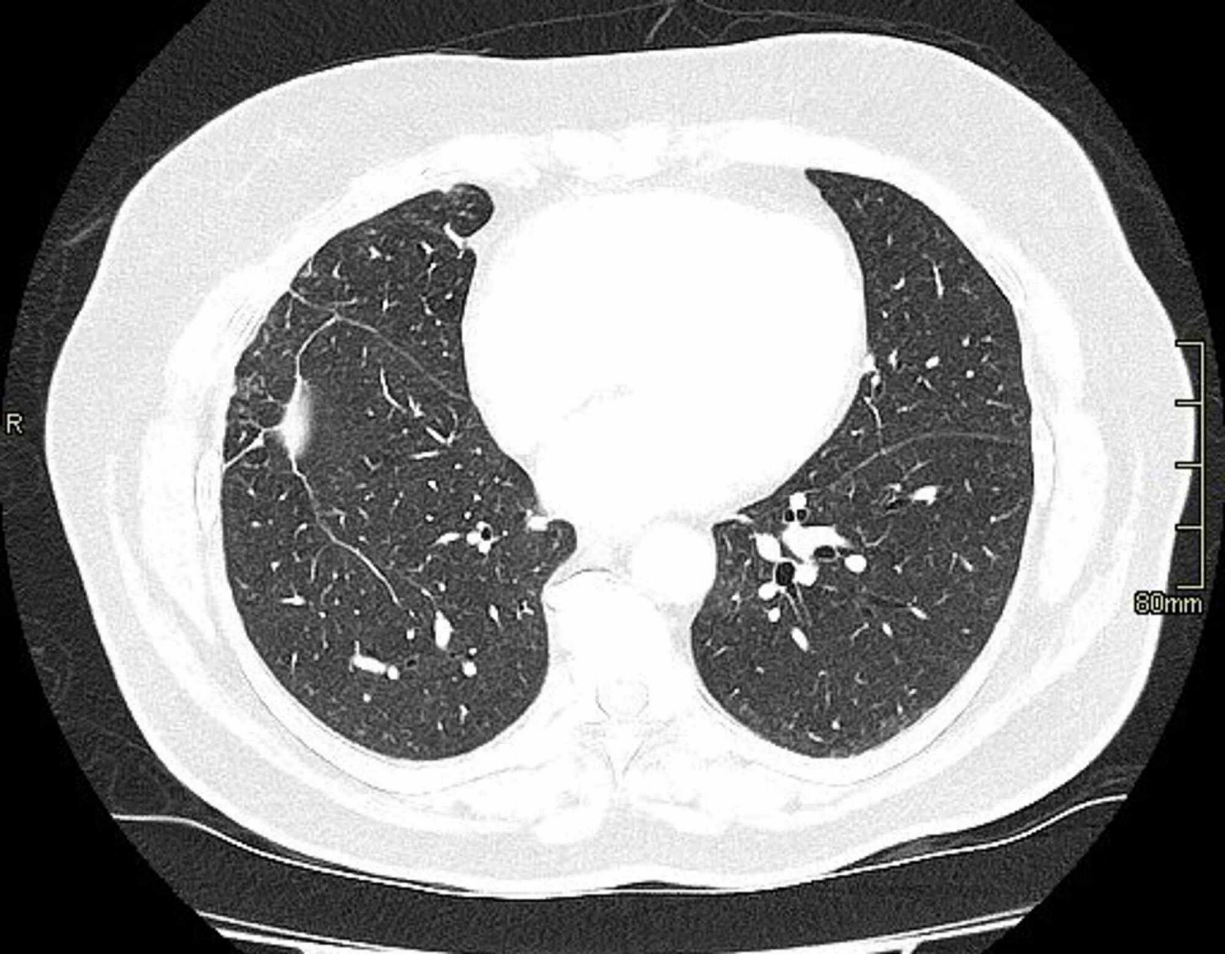
Resolution of ground glass changes after cessation of pembrolizumab.

Given the consistent ground glass changes on radiological studies, noncaseating granuloma on histological findings and the correlation in timeline with treatment, the diagnosis of pembrolizumab-related sarcoidosis-like granulomatous reaction was made. Pembrolizumab was stopped after discussion with the patient as there was evidence of systemic involvement. She was commenced on prednisolone 30 mg once a day orally and tapered over six weeks. She had marked improvement in her skin lesions and, repeat CT scan after eight weeks (Figure 4) showed resolution ground glass appearance. There was no evidence of disease progression. After eight weeks, follow up examination and restaging studies demonstrated stable disease and no evidence of skin lesions.

## Discussion

According to NICE guidelines [1], in adults with metastatic non-small cell lung cancer (NSCLC) which express PD-L1(at least 50% of tumor proportion score) and negative for epidermal growth factor, pembrolizumab is a recommended option. Several published trials have demonstrated the superiority of pembrolizumab over chemotherapy in patients with advanced NSCLC [2]. However, these treatments also have a wide range of side effects but sarcoid-like reactions are uncommon.

The term “sarcoid-like reaction” is usually used to describe localized reactions as compared to the systemic process seen in sarcoidosis. Yet, systemic involvement affecting the lungs, skin, and kidneys can be seen. Noncaseating granulomas are the hallmark of sarcoid-like reactions [3]. It is prudent to note that sarcoid-like reaction can masquerade as worsening or recurrence of cancer. In such cases, clinical presentation, coupled with appropriate biopsy, radiographic evidence of bilateral hilar lymphadenopathy (with paratracheal lymphadenopathy), and elevated serum ACE levels aid in distinguishing sarcoid-like reaction from recurrent or metastatic disease. In our patients, the diagnosis of the sarcoid-like reaction was suspected because of the typical pattern of skin lesion and noncaseating granuloma on biopsy and raised ACE levels. Sarcoid-like reactions are not limited to pembrolizumab but also associated with other checkpoint inhibitors, e.g. ipilimumab and nivolumab [4]. As these drugs are becoming standard of care for several malignancies, it is crucial for both oncologist and radiologists to be vigilant about sarcoid-like reaction as it is important for the appropriate management and possible measurement of therapeutic response.

At the cellular level, the interaction of monocyte-derived histiocytes and CD4+ Th1 cells is the plausible mechanism behind the pathogenesis of sarcoid reactions. Tumor necrosis factor-α (TNF-α) plays a central role not only in the formation but also in the maintenance of the “sarcoid” granulomata [5]. For example, anti-TNF-alpha monoclonal antibodies (i.e., infliximab and adalimumab) have been successfully used to treat sarcoidosis [6-7]. Paradoxically, some patients with inflammatory bowel disease, psoriasis, arthritis, or even sarcoidosis who received anti-TNF-α therapy developed sarcoidosis-like reactions to therapy [8]. These findings led the researcher to suggest that there is cytokine imbalance and promotion of Th1 immune response, which resulted from the impaired regulatory role of anti-TNF-α on autoreactive cells [9]. Either hypoactive or hyperactive immune response is responsible for sarcoidosis [10]. For instance, sarcoidosis unrelated to pembrolizumab has demonstrated hypoactive immune response with decreased T-cell proliferation and PD-1 up-regulation [11]. On contrary, there is a hyperactive immune response with increased T-cell proliferation and inhibition of PD-1 signaling in sarcoidosis related to immune-modulating drugs [9].

Sarcoidosis is a multi-systemic disease of unknown etiology and mostly a diagnosis of exclusion. A presumptive diagnosis of sarcoidosis is based on a constellation of clinical symptoms and radiographic features [12]. The hallmark radiological feature is bilateral hilar lymphadenopathy. In most cases, patients are asymptomatic or may present with erythema nodosum-like panniculitis [13]. Alternatively, the patient may present with a Heerfordet syndrome, the triad of uveitis, parotiditis, and fever. There may be an abnormal gallium imaging with increased uptake in the parotid and lacrimal gland and mediastinal nodes, i.e. paratracheal and bilateral hilar uptake [12].

As per guidelines for managing immune-mediated adverse effects, treatment depends on the severity of the disease [14]. For instance, asymptomatic cases can be managed by discontinuing and close monitoring, while corticosteroids should be reserved for systemic involvement or progression, while immunosuppressants can be added in severe cases [15]. Fortunately, the sarcoid-like reactions are reversible as per published cases. The adverse effects of our patient, for example, resolved with discontinuing of drug and commencement of steroids.

## Conclusions

Pembrolizumab-induced granulomatous sarcoid-like reaction is immune-mediated toxicity. It is prudent for both oncologists and radiologists to recognize such uncommon adverse effect of pembrolizumab as it can masquerade both clinically and radiologically as progression or recurrence of underlying cancer. Early diagnosis with the cessation of pembrolizumab can change the outcome of a clinical course for patients.
